# Prof. J. Boring

**Published:** 1868

**Authors:** 


### PROF. J. BORING.

We mentioned, in the February number of the JOURNAL, the election of Prof. JESSE BORING to the Chair of "Obstetrics and Diseases of Women and Children" in Atlanta Medical College, and his expected return from Galveston, Texas, to make Atlanta his permanent home. He arrived the first week of the Session; and we are pleased to find him full of energy and enthusiasm in the discharge of his duties in the Chair he so ably filled during the first three years's exercises of the College. Below we give the sentiments of respect and confidence, expressed by his late colleagues in Galveston, on accepting his resignation:

GALVESTON MEDICAL COLLEGE,
GALVESTON, TEXAS, March 14, 1868.

PROF. J. BORING—Sir: We have the honor to hand you herewith a copy of the resolutions which were introduced, and unanimously adopted by the Faculty, upon receiving your resignation as a Professor in this College.

Very respectfully, your obedient servants,

GREENSVILLE DOWELL, M.D., Prof. Surgery,
T. J. HEARD, M.D., Prof. Theory & Practice, } Com'tee.
F. E. DANIEL, M.D., Prof. Anatomy,

COPY OF RESOLUTIONS

adopted by the Faculty of the Galveston Medical College, at a meeting held March 13th, 1868 :

*Mr. President, and Gentlemen of the Faculty:*

Your Committee, appointed "to draft resolutions expressive of our sentiments in severing the relations which have heretofore existed between Professor Boring and this College," beg leave respectfully to submit the following:

"WHEREAS, Professor Jesse Boring, the Founder and President of the Galveston Medical College, has tendered his resignation as Professor of Obstetrics; and Whereas, said resignation has been accepted, by this Faculty, therefore,

*Resolved*, That it is with feelings of no ordinary regret that we are compelled, out of consideration for the wishes of Professor Boring, and of the motives which prompted him to offer his resignation, to consent to his retirement from the Faculty; and that we do so in the hope that the change which he proposes to make may promote the health and happiness of himself and family.

*Resolved*, That in parting with Professor Boring, the Galveston Medical College loses one of its ablest teachers, and sustains a loss which is indeed irreparable; that we, as a Faculty, lose an esteemed friend and a colleague whose absence will be seriously felt in our deliberations and in the social circle, as well as in the lecture room; and this community will deplore the loss of one of its best citizens, and most exemplary members of society.

*Resolved*, That these resolutions be spread upon the records of the College, and that a copy be handed Professor Boring, with the expression of the sincere hope that in returning to his old home in Georgia, his hopes and expectations may be most fully realized, and that he may find friends there who will appreciate and prize as we do, his many excellencies as a Christian gentleman, a citizen, a friend, and a teacher and practitioner of medicine.

Respectfully submitted,

GREENSVILLE DOWELL, M.D., Prof. Surgery,<br>

T. J. HEARD, M.D., Prof. Theory & Practice,<br>

F. E. DANIEL, M.D., Prof. Anatomy,<br>

